# Reduced FANCE Confers Genomic Instability and Malignant Behavior by Regulating Cell Cycle Progression in Endometrial Cancer

**DOI:** 10.7150/jca.86348

**Published:** 2023-08-28

**Authors:** Chunying Zheng, Zhen Ren, Hongliang Chen, Xiaorui Yuan, Suye Suye, Huan Yin, Chun Fu

**Affiliations:** Department of Obstetrics and Gynecology, The Second Xiangya Hospital of Central South University, Changsha, Hunan, 410011, China.

**Keywords:** endometrial cancer, FANCE, genomic instability, cell cycle, proliferation, chemoresistance

## Abstract

**Introduction:** Fanconi anemia complementation group E (FANCE) is a subunit of fanconi anemia (FA) pathway and plays a key role in repairing DNA interstrand cross-links (ICLs) damage. We investigate detailed functions and mechanisms of FANCE in endometrial cancer (EC).

**Methods:** FANCE protein and RNA expression in EC and non-cancerous tissues were detected by Western blotting (WB), immunohistochemistry (IHC), and real-time polymerase chain reaction (RT-PCR) assays. Using lentiviral transfection and siRNA interference techniques, we constructed overexpressing FANCE (OE-FANCE) and FANCE-knockdown (FANCE-KD) EC cells. We then investigated DNA damage repair capacity of FANCE in EC cells including comet assay and γH2AX immunofluorescence assay. *In vitro* assays including CCK8, EDU and colony formation for chemoresistance and proliferation, transwell assay for metastasis were performed. Flow cytometer assay, cell cycle synchronization for cell cycle progression and EC cells RNA sequencing were determined. Finally, *in vivo* mouse models were used to detect tumor growth.

**Results:** We found FANCE RNA and protein expression was significantly decreased in endometrioid adenocarcinoma (EAC) compared with normal and atypical hyperplasia endometrium. FANCE promoted the repair of ICL damage and double-strand break (DSB) in OE-FANCE EC cells. Furthermore, FANCE increased drug resistance in OE-FANCE EC cells by upregulating FA pathway and homologous recombination (HR) associated proteins. FANCE inhibited cell proliferation and metastasis through G2/M cell cycle arrest *in vitro* and vivo. FANCE participated in regulating several pathways.

**Conclusion:** The study demonstrates the reduction of FANCE expression leads to genomic instability, thereby promoting the development of EC by regulating cell cycle.

## Introduction

Endometrial cancer (EC) is one of the most common gynecological malignancies worldwide with 417367 new cases and 97370 deaths in 2020^1^. EC ranks second among female reproductive system malignancies in developing countries, and morbidity and mortality continue to rise^2^. EC is divided into two histological types according to the Bokhman classification^3^. Endometrioid adenocarcinoma (EAC) originates from the endometrial glandular epithelium and belongs to type I EC, accounting for 80-90% of EC^4^. The development of EC is a multiple process of accumulating genetic and epigenetic mutations. Next-generation sequencing analysis reveals homologous recombination DNA damage repair (HR-DDR) deficiencies in EC are the most common in all solid tumors^5^. Among DNA damage repair pathways, mismatch repair pathway is the most widely studied in EC. Patients with mismatch repair deficiency have a moderate prognosis^6^. However, there are few studies on the roles of other DNA damage repair pathways in EC.

Fanconi anemia (FA) is a syndrome of genomic instability that leads to aplastic anemia, dysplasia, and predisposition to hematological and solid organ malignancies^7^. The proteins encoded by 22 FA genes are involved in the DNA repair pathway, known as the "FA pathway". The FA pathway is primarily responsible for repairing DNA interstrand cross-link (ICL) damage and detecting genomic integrity throughout the cell cycle^8^. Furthermore, the FA pathway is involved in the regulation of gene transcription, apoptosis and chemoresistance^9^, ^10^. Fanconi anemia complementation group E (FANCE) encodes a protein of 536 amino acids located in the nucleus^11^. FANCE is an important part of the FA core complex and acts as a bridge between the FA core complex and FANCD2, mediating the activation of the FA pathway^12^. Related studies on FANCE have shown that mutations in FANCE are associated with the incidence of esophageal squamous cell carcinoma (ESCC) in Iran and China^13^, ^14^. High expression of FANCE in hepatocellular carcinoma (HCC) may promote tumor proliferation by activating cell cycle signaling, and be a biomarker of poor prognosis^15^. At present, the role of FANCE in EC has not been investigated and further research may be meaningful.

This study first investigated the expression of FANCE in EAC and non-cancerous tissues, and its correlation with clinicopathological factors of EAC patients. A series of *in vitro* experiments were then carried out by lentivirus overexpression and siRNA knockdown of FANCE in EC cells to examinate DNA damage repair, drug resistance, cell cycle and proliferation. A nude mouse subcutaneous tumorigenesis model was constructed to confirm the effect of FANCE on proliferation. The research aims to reveal the role of FANCE in the occurrence and development of EC. The results may provide a new theoretical basis for the pathogenesis of EC.

## Materials and Methods

### Clinical patient specimens

Approval for this work was obtained from the Research Ethics Committee of Second Xiangya Hospital, Central South University (No.2017-032). All patients obtained the informed consent. Eighty-three newly diagnosed EAC cases were collected between 2020 and 2021. All EAC patients received initial surgery treatment. The inclusion and exclusion criteria are listed as follows: The inclusion criteria were: (1) postoperative pathology was endometrioid adenocarcinoma; (2) patients with complete clinical data. The exclusion criteria were: (1) patients combined with other EC pathology; (2) patients combined with other malignant tumors; (3) patients receiving chemotherapy or radiotherapy or other treatment before surgery. Thirty atypical endometrial hyperplasia (AEH) paraffin tissues were collected. Eighty-three normal endometrium tissues were collected from cases of cervical intraepithelial neoplasia. EAC fresh tissues, normal endometrium tissues, paired adjacent normal tissues were collected for RNA and protein analysis. The adjacent normal tissue was taken >3 cm distance from the tumor lesion margin and no abnormal cells assessed by pathology^16^.

We collected various clinical information for EAC patients, including patient age, menstrual status, tumor histological grade, tumor stage, depth of myometrial invasion (MI), lymph node metastasis status, lympho-vascular involvement (LVI) status, mismatch repair (MMR) status of tumor tissue, P53 and Ki-67 levels of tumor cells. The tumor stage was referred to the 2009 International Federation of Gynecology and Obstetrics (FIGO) guidelines^17^. We collected age at diagnosis, hypertension, diabetes, menopause and family history of malignancy for EAC, AEH and normal endometrium patients.

### Tissue immunohistochemistry (IHC)

All paraffin-embedded sections were deparaffinized, rehydrated, and blocked to endogenous peroxidase activity. After antigen retrieval, the slides were incubated with goat blocking serum for 10 min and then incubated with FANCE rabbit polyclonal primary antibody (Biobryt, orb156841) diluted to 1:200 at 4℃ overnight. The secondary antibody kit (ZSGB-BIO, PV-9000) and 3,3-Diaminobenzidine (DAB) chromogenic kit (ZSGB-BIO, ZLI-9018) were used the next day. Carl Zeiss AG microscope was used to capture the images at 100× magnification.

Two pathologists evaluated the staining independently. The staining extent was graded based on the product of staining intensity and staining percentage. Stain intensity was defined as 0 (negative), 1 (weak), 2 (moderate), 3 (strong). Stain percentage was defined as 0 (< 25%), 1 (25%~49%), 2 (50%~74%), 3 (75%~100%). For IHC statistical analysis, stain index ≤3 was classified as FANCE-low expression and stain index >3 was FANCE-high expression^18^.

### Cell culture

Endometrial cancer cell lines HEC-1-A (Procell, CL-0099) and HEC-1-B (Procell, CL-0100) were purchased from Procell Life Science&Technology Co., Ltd. The cell lines were genotyped and authenticated at the beginning of the experiments. HEC-1-A were cultured in McCoy's 5A medium (BOSTER, PYG0026) and HEC-1-B were cultured in minimum essential medium (MEM, Gibco). Each medium was supplemented with 10% fetal bovine serum (FBS, BI) and 1% penicillin-streptomycin (Gibco) and cultured at 37 °C in a humidified incubator with 5% CO_2_.

### Lentivirus infection and RNA interference

Lentivirus overexpressing FANCE (OE-FANCE) and negative control (NC) were used to infect cells, and puromycin was used to select stably transfected cells. The small interfering RNAs (siRNAs) oligonucleotide sequences used to knockdown FANCE in EC cells were 5'- GCUUCUCCACUGUCUGAAATT-3' (forward), 5'- UUUCAGACAGUGGAGAAGCTT-3' (reverse). The negative control sequences were 5'-UUCUCCGAACGUGUCACGUTT-3' (forward) and 5'-ACGUGACACGUUCGGAGAATT-3' (reverse). Cells were transfected with a mixture of siRNA and Lipofectamine 2000 reagent (Invitrogen) in serum-free medium for 24 h.

### RNA extraction and qRT-PCR analysis

Total RNA was extracted from human tissues and cell lines using RNAiso Plus reagent (Takara, Code No.9108) and was reverse transcribed into cDNA using a Reverse Transcription kit (Vazyme, Q711-02). SYBR Green Master Mix kit (Vazyme, R323-01) was used for qRT-PCR to detect the expression of FANCE and the housekeeping gene glyceraldehyde 3-phosphate dehydrogenase (GAPDH). The primers used for RT-PCR were as followed: FANCE, 5'- CTCGGTCTCCTGCGGCTCTG -3' (forward), and 5'- GGCGGGAGGCTGAGGAAGTC -3' (reverse); GAPDH, 5'- CATCACTGCCACCCAGAAGACTG-3' (forward), and 5'- ATGCCAGTGAGCTTCCCGTTCAG-3' (reverse). 2^-ΔΔCt^ method was performed to calculate the relative expression of the mRNA.

### Western blotting (WB) assay

The tissue and cell proteins were isolated using radio immunoprecipitation assay (RIPA) lysis buffur (Epizyme Biotech) supplemented with protease inhibitor. The Bradford method (CWBIO) was used to determine the protein concentrations. Proteins were electrophoresed on Sodium dodecylsulfate-polyacrylamide gel electrophoresis (SDS-PAGE, CWBIO), then transferred to polyvinylidene fluoride (PVDF) membranes, blocked, and incubated with primary anti-FANCE (Biobryt, 1:10000), anti-FANCD2 (1: 5000; Novus Biologicals), anti-GAPDH (1:10000, bioworld), anti-β-tubulin (1:10000, proteintech), anti-RAD51(1:5000, Abcam), anti-γH2AX (1:10000, Abcam), anti-cyclin dependent kinase 4 (CKD4) (1:5000, proteintech), anti-cyclin dependent kinase 6 (CDK6) (1:5000, proteintech), anti-cyclin D1 (1:5000, proteintech), anti-cyclin B1(1:5000, proteintech), anti-cyclin E(1:5000, proteintech) and anti-cyclin A2(1:5000, proteintech) for overnight at 4℃. Secondary antibodies were incubated for 2 h the next day. The protein bands on the membranes were detected using Enhanced Chemiluminescent (ECL, NCM Biotech).

### CCK-8 assay and drug treatment

HEC-1-A cells were plated at a concentration of 3×10^3^ cells per well and HEC-1-B cells were plated at a concentration of 4×10^3^ cells per well in 96-well plates for cell proliferation assays. After 6 h of adherence, we added 10ul of Cell-Counting-Kit-8(CCK8, AbMole) reagent and incubated the cells for 2 h at 37℃, and read the absorbance at 450nm. We record the optical density (OD) values at 0 h, 24 h, 48 h and 72 h.

The inhibitory concentrations 50% (IC50) values were determined. Cells were collected and seeded in 96-well plates at 8000 per well in culture medium containing Cisplatin (MCE, CAS No.15663-27-1) (0, 2.5, 5, 10, 15, 20 μM) or mitomycin C (MMC; Sigma, CAS No.1082532-95-3) (0, 0.625, 1.25, 2.5, 5, 10 μM). Cisplatin/MMC sensitivity in EC cells was determined using CCK-8 assay.

### EDU fluorescent microscopy

EC cells were seeded in 96-well plates at a density of 4000 cells/well for 24 h. 10uM 5-ethynyl-2'deoxyuridine (EDU) regent was added and incubated each well for 2 h. Click Addictive Solution were treated for 30 min and Hoechst 33342 treated for 10 min. For each well, five fields were randomly captured at 200× magnification to count the average number of EDU foci per nucleus.

### Colony formation assay

Five hundred OE-FANCE cells were split per well in 6-well plates and cultured for fifteen days. Two thousand FANCE-KD cells were split per well in 6-well plates. After seven days culture, cells were fixed in 4% paraformaldehyde for 30min and were stained with 0.5% Giemsa. Finally, we captured the images and counted the blue colonies.

### Transwell assay

24-well plates with 8-μm pore polycarbonate membranes (Corning 3422) precoated with Matrigel (Corning 356234) were used. A total of 5×10^4^ EC cells were suspended in 250μl serum-free medium and seeded into the upper chamber. And 750μl medium with 20% FBS was added into the lower chambers as a chemoattractant. After incubation for 48h at 37℃, invaded cells were fixed with 4% paraformaldehyde for 30min and stained with 0.5% Giemsa. Scrub the cells in upper chamber and capture.

### Cell cycle assay and synchronization treatment

Cells were collected and fixed overnight at 4℃ with 70% ethanol. The fixed cells were stained dark at 37℃ for 30 min with buffer saline (PBS) containing 50 μg/ml propidium iodide (PI, Beyotime, C1052) and 100 μg/ml RNase A (Beyotime, ST576). Beckman A00-1-1102 flow cytometer was used to analyze the percentage of each cell cycle on the PI fluorescence histogram. Synchronization of cell cycle was by thymidine double blockade^19^. All cells were simultaneously released from early S phase and protein was isolated at 0, 2, 4, 6, 8, 10, 12 h. The expression of cell cycle regulators was detected by WB.

### Immunofluorescence assay

Two thousand cells were planted on 24-well plate round coverslips, and then untreated or treated with 5 μM cisplatin for 24 h. Cells were fixed, permeabilized, then blocked with 10% goat serum in PBS for 30 min at room temperature, the cells were incubated overnight with γ-H2AX (1:200) primary antibodies at 4 °C. The next day, cells were incubated with fluorescent secondary antibody for 1 h at room temperature. We removed the round coverslip and inverted it on the DAPI-added slide. One hundred fields of view were randomly counted using an oil lens. Quantification of the γ-H2AX foci fluorescence was measured with Image J software.

### Comet assay

Four thousand OE-FANCE cells and corresponding NC cells were seeded on 6-well plate, and untreated or treated with 5μM cisplatin for 1 h. Then cells were washed with PBS, incubated with complete medium for 6 h, and untreated or treated with 4 nM bleomycin sulfate (BLEO) for 30 min. According to comet assay (Abcam) instructions, cells were embedded in soft agarose on glass slides and cleaved under alkaline conditions. Next, cells were electrophoresed and stained with Vista Green DNA Dye. Images of 100 cells for each group were captured with Carl Zeiss AG fluorescence microscope at 100× magnification. Comet Assay Software Project program was used to quantify tail moment.

### Cellular RNA sequencing analysis

Three biological replicates of 1×10^7^ HEC-1-A OE-FANCE and HEC-1-A NC cells were collected using TRIzol reagent. Cell total RNA was extracted and quantified and qualified. The clustering of the index-coded samples was performed on a cBot Cluster Generation System using TruSeq PE Cluster Kit v3-cBot-HS (Illumia) according to the manufacturer's instructions. After cluster generation, the library preparations were sequenced on an Illumina Novaseq6000 platform and 150 bp paired-end reads were generated. GSEA analysis was conducted and FDR (q-value) < 0.25 and p < 0.05 were considered statistically significant.

### *In vivo* tumorigenicity assay

The experimentation was approved by the Experimental Animal Ethics Committee of the Second Xiangya Hospital of Central South University (No. 2020388). Sixteen female athymic nude mice were purchased from Hunan SJA laboratory animal Co., Ltd and maintained in Animal Experiment Center of Science and Education Building of Second Xiangya Hospital in accordance with Regulations on the Administration of Laboratory Animals. 1×10^6^ HEC-1-A OE-FANCE and HEC-1-A NC cells were injected subcutaneously into the right dorsal flank at 5 weeks old, respectively. The formation of subcutaneous tumors was recorded as the first day, followed by measurement of tumor volume and weight in nude mice every other day. Nude mice were executed on day 23 using the cervical dislocation method and tumors were photographed *in vivo* and post-dislocation images. The tumor volume was calculated using the following formula: volume = length × width ^2^/2 (length = longest diameter, width = corresponding vertical diameter). Ultimately, tumor volume and weight profiles of nude mice were plotted over time.

### HE staining

Tumor tissues were fixed and embedded in paraffin. The samples were sectioned at 5 μm thickness and stained with hematoxylin and eosin (H&E). The expression levels of Ki-67 (Abcam, ab92742) and FANCE in implanted tumor of mice were evaluated by IHC, according to the previously described method above.

### Statistical analysis

SPSS26.0 was used for statistical analysis. Chi-square test was used for FANCE differential expression between three histopathological categories and clinical data comparison between FANCE-high and FANCE-low group in EC. Bonferroni's method was used for pairwise comparisons between the three histopathological categories. Paired t-test was used for FANCE expression of tissue paired samples. In addition, the results of cell functional experiments were analyzed by Student's t test. The continuous variable was shown as the mean ± standard deviation (SD). GraphPad Prism software was used for statistical charts. P< 0.05 (* p < 0.05, ** p < 0.01 and *** p < 0.001) was considered significant.

## Results

### FANCE expression in EAC tissues and non-cancerous tissues

**Figure 1:**
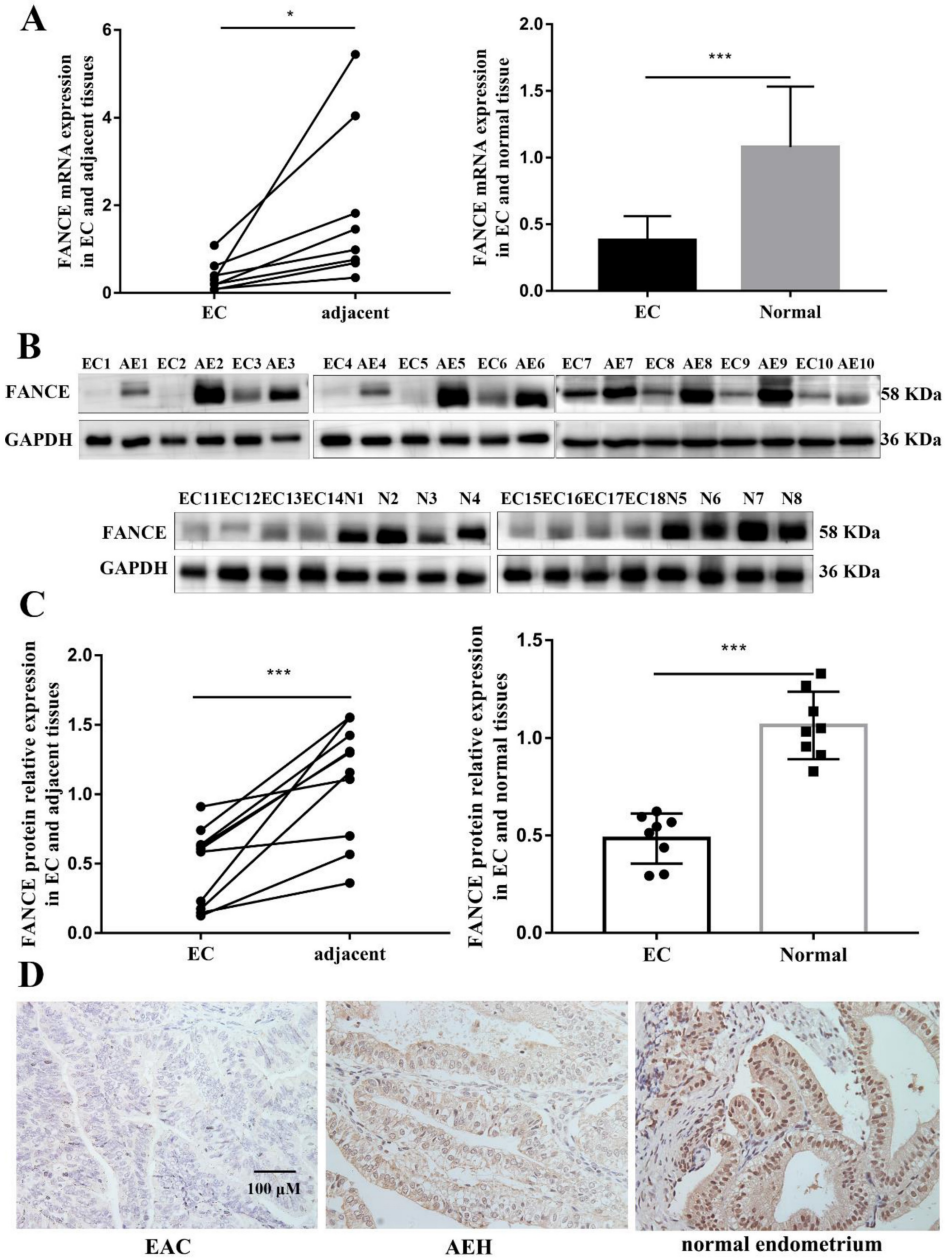
** FANCE RNA and protein expressions in endometrioid adenocarcinoma (EAC) and non-cancerous tissues** (A) FANCE RNA expression was significantly downregulated in EAC compared to adjacent endometrium(n=8) and normal endometrium tissues(n=10). (B) FANCE protein expression in EAC, adjacent endometrium(n=10) and normal endometrium tissues(n=8). (C) FANCE protein expression was significantly downregulated in EC compared to adjacent endometrium and normal endometrium tissues. (D) IHC FANCE expression in EAC, atypical endometrial hyperplasia and normal endometrium tissues. Scale bar, 100μM. * p < 0.05, *** p < 0.001.

In order to determine the clinical significance of FANCE in EAC, FACNE RNA and protein expression were detected in EAC and non-cancerous tissues. Both FANCE mRNA and protein expression were decreased significantly in EAC tissues as compared with corresponding adjacent endometrium tissues and normal endometrium (Fig. 1A-C).

The progression of the endometrium from beyond the normal proliferative stage to EAC is a continuous process in which atypical hyperplasia is classified as a precancerous lesion. Subsequently, we examined whether FANCE continued to be significantly downregulated with pathological progression. We analyzed immunohistochemical expression of FANCE in 83 normal endometrium tissues, 30 AEH tissues and 83 EAC tissues (Fig. 1D). There was no statistically significant difference in age at diagnosis, hypertension, diabetes, menopause and family history of malignancy. FANCE-low expressions were 13.3%, 20% and 50.6% in normal endometrium, AEH and EAC respectively. The IHC results showed that the expression of FANCE was significantly lower in EAC than in normal endometrium (p<0.05) and AEH (p<0.05). There was no difference in expression between normal endometrium and AEH (Table 1). The correlation analysis between the expression of FANCE protein and clinicopathological characteristics revealed decreased expression of FANCE was associated with older age (p=0.021) and high tumor grade (p=0.035) (Table 2).

### FANCE promoted the repair of ICL damage in overexpressing FANCE EC cells

**Figure 2:**
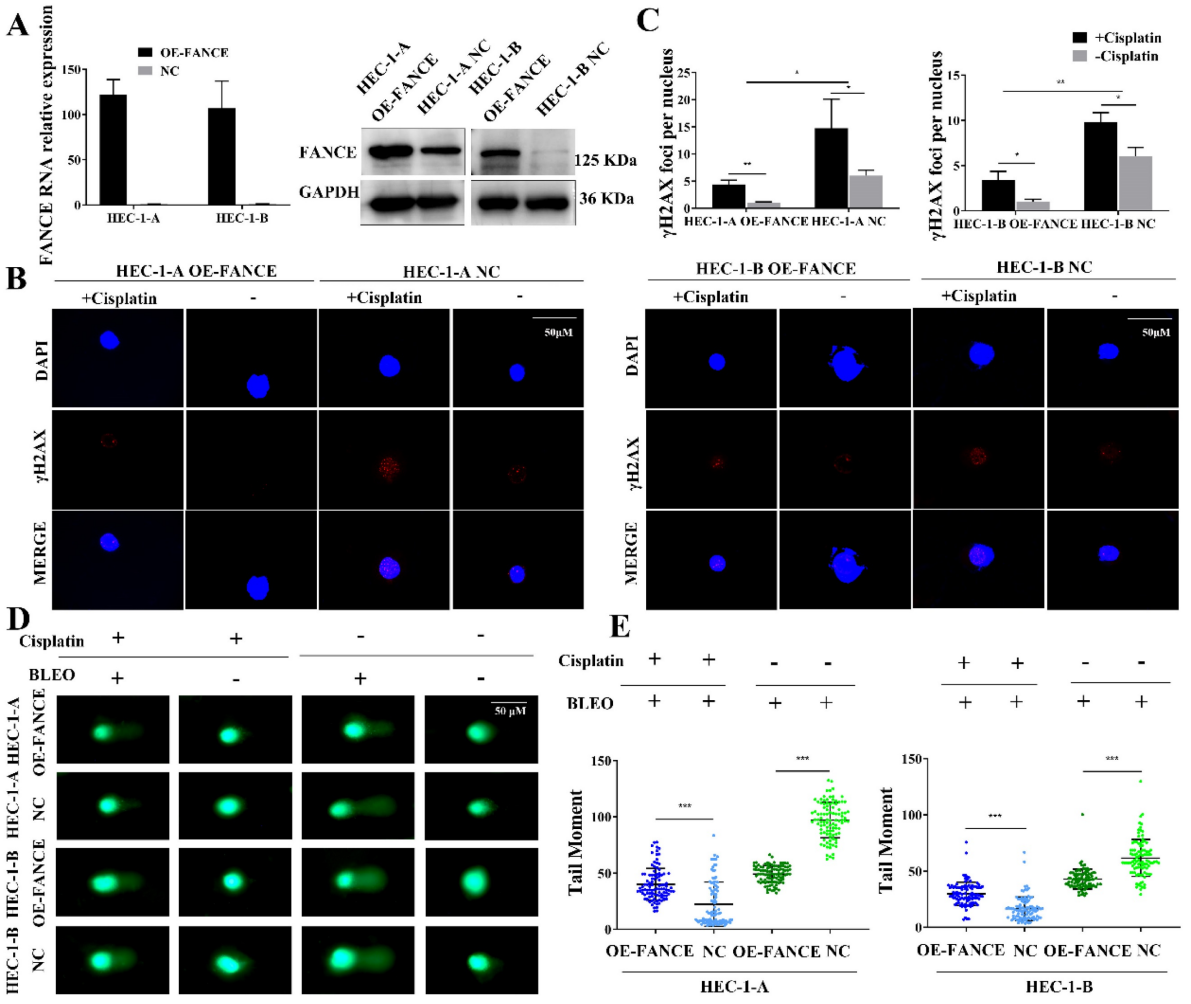
** FANCE promoted the repair of ICL induced DSB damage in EC cells** (A) The efficiency of overexpressing FANCE (OE-FANCE) with lentiviral transfection in HEC-1-A and HEC-1-B cells was verified. (B) γH2AX foci (red) in EC cells was detected following treatment with cisplatin (5μM) for 24 h. The nucleus staining was blue. Scale bar, 50μM. (C) Bar chart showing γH2AX foci was significantly decreased in EC OE-FANCE cells. Cisplatin treatment induced an increase in γH2AX foci. n > 100 cells/treatment. (D) The comet tail moment following treatment with cisplatin(5μM) for 1h, BLEO(4nM) for 30 min or their combination. Scale bar, 50μM. (E) The Scatter plot showing the comet tail moment of HEC-1-A and HEC-1-B OE-FANCE was decreased compared to NC group when treatment with BLEO (4nM) for 30 min. Cisplatin (5 μM) for 1 h and followed by BLEO (4 nM) for 30 min. The tail moment of EC cells was reduced, and the reduction of HEC-1-A and HEC-1-B NC cells was more pronounced. * p < 0.05, ** p < 0.01 and *** p < 0.001.

To evaluate whether FANCE plays a role in the repair of ICL induced double-strand break (DSB) damage in EC, we determined γH2AX expression and DNA breakage level. FANCE expression in HEC-1-A overexpressing FANCE (OE-FANCE) cell, HEC-1-B OE-FANCE cell and corresponding negative control (NC) cell was confirmed (Fig. 2A). In response to ICL agents such as cisplatin and MMC, the histone protein H2AX is phosphorylated to form γH2AX foci. As shown in Fig. 2B, cisplatin induced increased γH2AX foci after a 24 h-treatment in HEC-1-A NC cells. Compared to HEC-1-A NC cells, HEC-1-A OE-FANCE exhibited increased DNA repair through significantly reducing γH2AX staining (Fig. 2C). The results were the same in HEC-1-B OE-FANCE and NC cells.

We directly detected cellular DNA damage by comet assay using cisplatin as a DNA ICL agent and BLEO as a DNA fragmentation agent (Fig. 2D, E). None of the EC cells formed distinct comet tails when treatment-free or cisplatin treatment. After BLEO treatment, the DSB repair ability of HEC-1-A OE-FANCE cells was enhanced, and the comet tail distance was significantly lower than that of HEC-1-A NC cells. When treated with cisplatin followed by BLEO treatment, the comet tail distance of all cells was shortened. The enhanced ability of HEC-1-A OE-FANCE cells to repair ICL resulted in less shortened comet tail distance compared with HEC-1-A NC cells. The results were the same in HEC-1-B OE-FANCE and NC cells.

### FANCE increased drug resistance in OE-FANCE EC cells by upregulating FA pathway

**Figure 3:**
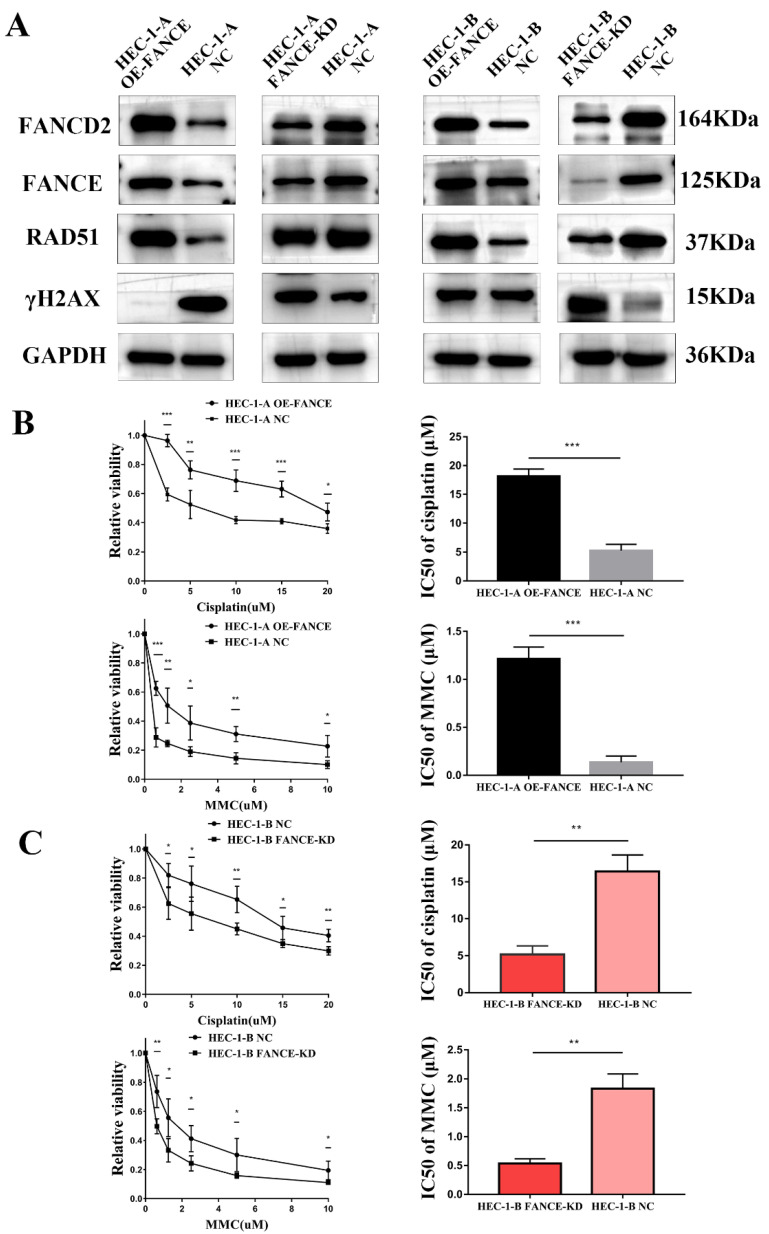
** Overexpression of FANCE mediated increased drug resistance by upregulating FA pathway** (A) WB analysis showed OE-FANCE induced increased expression of FANCD2, RAD51, and decreased expression of γH2AX in EC cells. FANCE-KD induced the opposite expression. GAPDH was used as the housekeeping protein. (B) EC cells were incubated with cisplatin or MMC at a range of concentrations for 48 h, and cell viability was measured by CCK8 assays. GraphPad Prism 7.0 was used to calculate the IC50 of the drug. The cisplatin IC50s of HEC-1-A OE-FANCE and HEC-1-A NC cells were 19.477μM and 5.792μM respectively. The MMC IC50s of HEC-1-A OE-FANCE and HEC-1-A NC cells were 1.334μM and 0.095μM. (C) The cisplatin IC50s of HEC-1-B FANCE-KD and HEC-1-B NC cells were 6.21μM and 14.564μM. The MMC IC50s of HEC-1-B FANCE-KD and HEC-1-B NC cells were 0.562μM and 1.799μM. * p < 0.05, ** p < 0.01 and *** p < 0.001.

We further explored the changes in the FA and HR pathways of EC cells after interference with FANCE and the influence on chemoresistance. We simultaneously constructed HEC-1-A FANCE-knockdown (FANCE-KD) cell, HEC-1-B FANCE-KD cell, and corresponding negative control (NC) cell. We detected the expression of FA pathway-associated protein FANCD2, HR pathway protein RAD51, and γH2AX in EC cells. In HEC-1-A OE-FANCE and HEC-1-B OE-FANCE cells, FANCD2 and RAD51 were significantly increased, while γH2AX was significantly decreased. The opposite trend was seen in FANCE-KD EC cells (Fig. 3A).

Compared with HEC-1-A NC, the IC50 of cisplatin and MMC treatment of HEC-1-A OE-FANCE cells were significantly higher, indicating a decrease in chemosensitivity (Fig. 3B). While HEC-1-B FANCE-KD cells exhibited increased chemosensitivity with a significantly decreased IC50 of cisplatin and MMC (Fig. 3C). The results demonstrated FA pathway upregulation mediated chemoresistance in EC.

### FANCE inhibited cell proliferation and invasive ability in OE-FANCE EC cells

**Figure 4:**
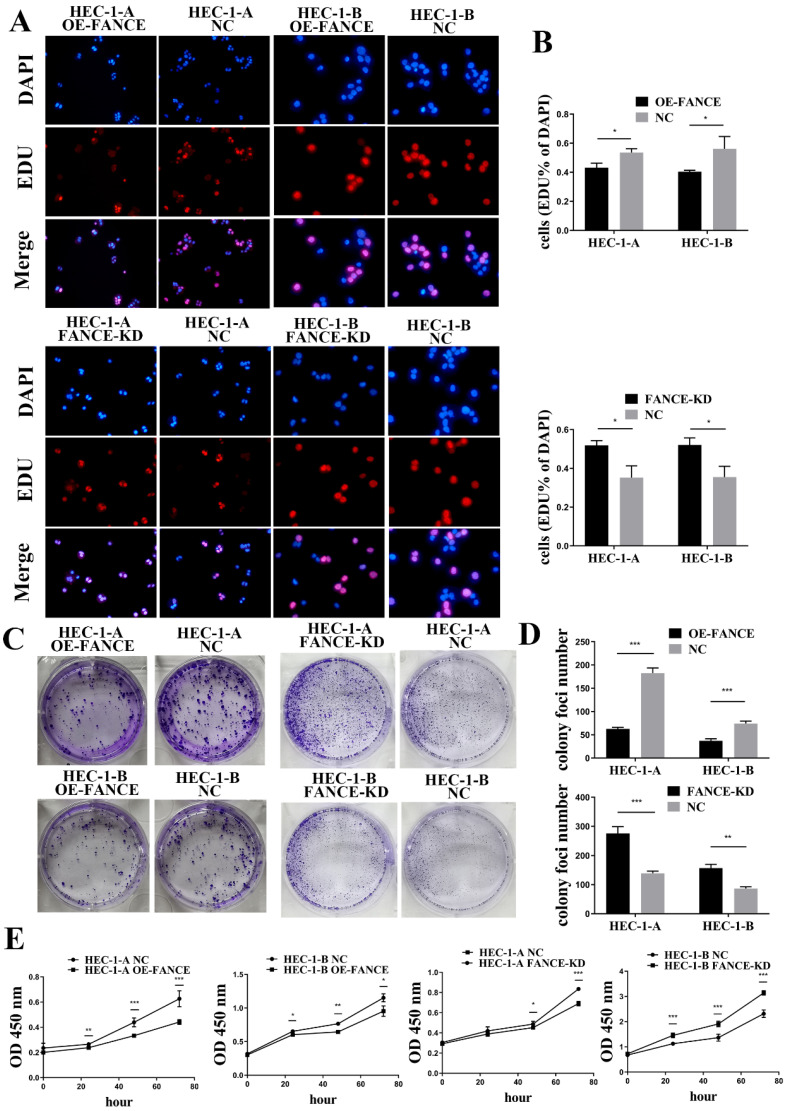
** FANCE inhibited proliferation ability of EC cells** (A) The proliferating cells were red using EDU staining. Nucleus was blue under DAPI staining. (B) Bar chart showed proliferative capacity was suppressed in HEC-1-A OE-FANCE and HEC-1-B OE-FANCE cells. (C) Representative images of colonies formation assays. (D) Bar chart showed colony formation capacity was suppressed in HEC-1-A OE-FANCE and HEC-1-B OE-FANCE cells. (E) CCK8 assay showed proliferative capacity was suppressed in HEC-1-A OE-FANCE and HEC-1-B OE-FANCE cells. HEC-1-A FANCE-KD and HEC-1-B FANCE-KD cells showed the opposite result. * p < 0.05, ** p < 0.01 and *** p < 0.001.

**Figure 5:**
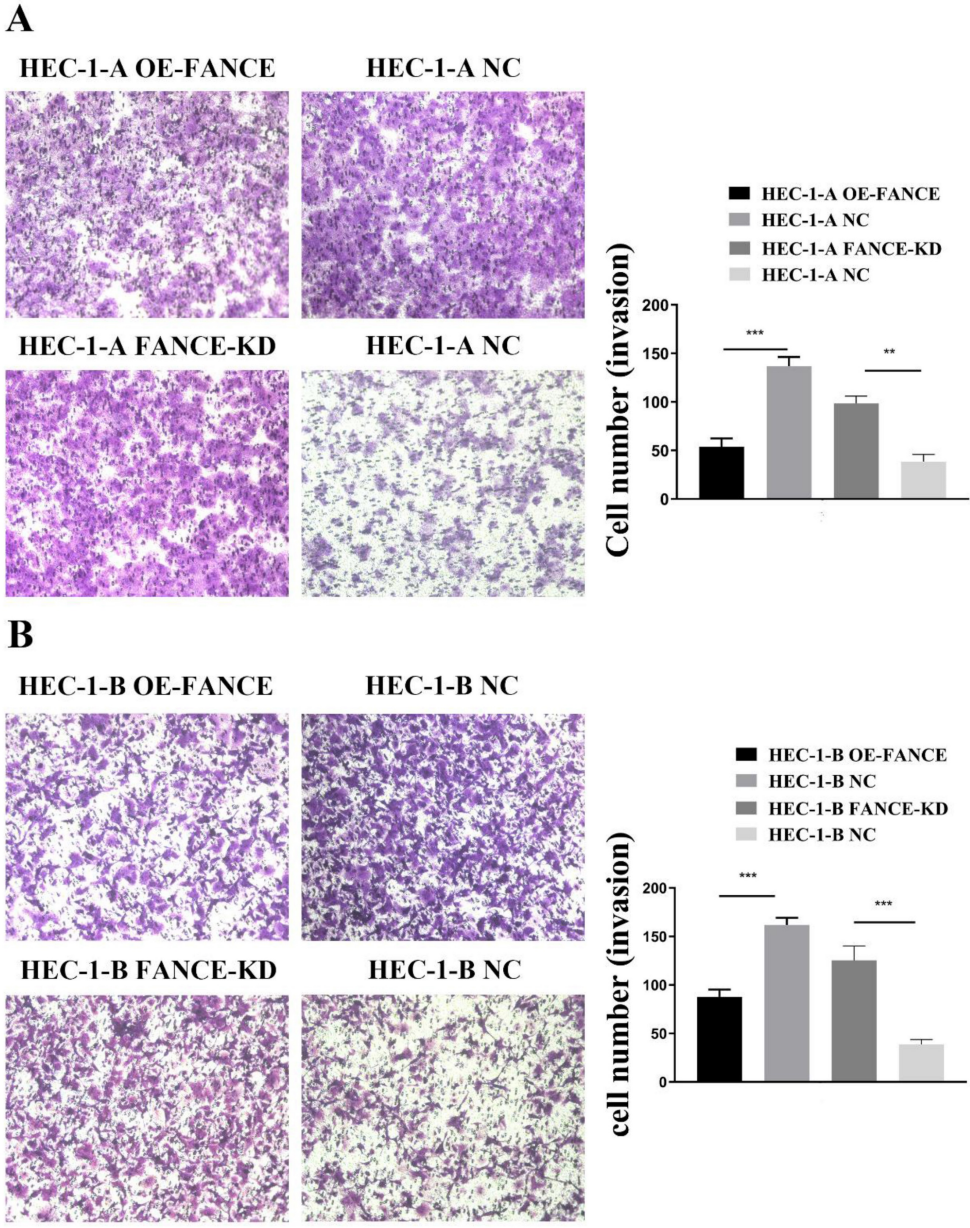
** FANCE inhibited invasive ability of EC cells** (A) Images of transwell assay of HEC-1-A and OE-FANCE and FANCE-KD cells and corresponding NC cells. Bar chart showed invasion capability was suppressed in OE-FANCE cells (B) Images of transwell assay of HEC-1-B and OE-FANCE and FANCE-KD cells and corresponding NC cells. Bar chart showed invasion capability was suppressed in OE-FANCE cells. ** p < 0.01, *** p < 0.001.

Immortal proliferation, invasion and metastasis are the most common malignant phenotypes of tumor cells. We examined the effect of FANCE on the proliferation and invasive capacity of EC cells *in vitro*. As shown in Fig. 4A-B, the cell proliferation rates of HEC-1-A OE-FANCE and HEC-1-B OE-FANCE cells were significantly lower than HEC-1-A NC and HEC-1-B NC cells by EDU assay, respectively. Increased expression of FANCE suppressed the number and size of clonal colonies in HEC-1-A and HEC-1-B cells (Fig. 4C, D). Consistently, HEC-1-A and HEC-1-B OE-FANCE cell viability was significantly reduced as assessed by the CCK-8 assay. Moreover, HEC-1-A OE-FANCE and HEC-1-B OE-FANCE cells showed reduced invasive ability (Fig. 5). The opposite results were shown in HEC-1-A FANCE-KD and HEC-1-B FANCE-KD cells.

### FANCE suppressed cell cycle progression in OE-FANCE EC cells

**Figure 6:**
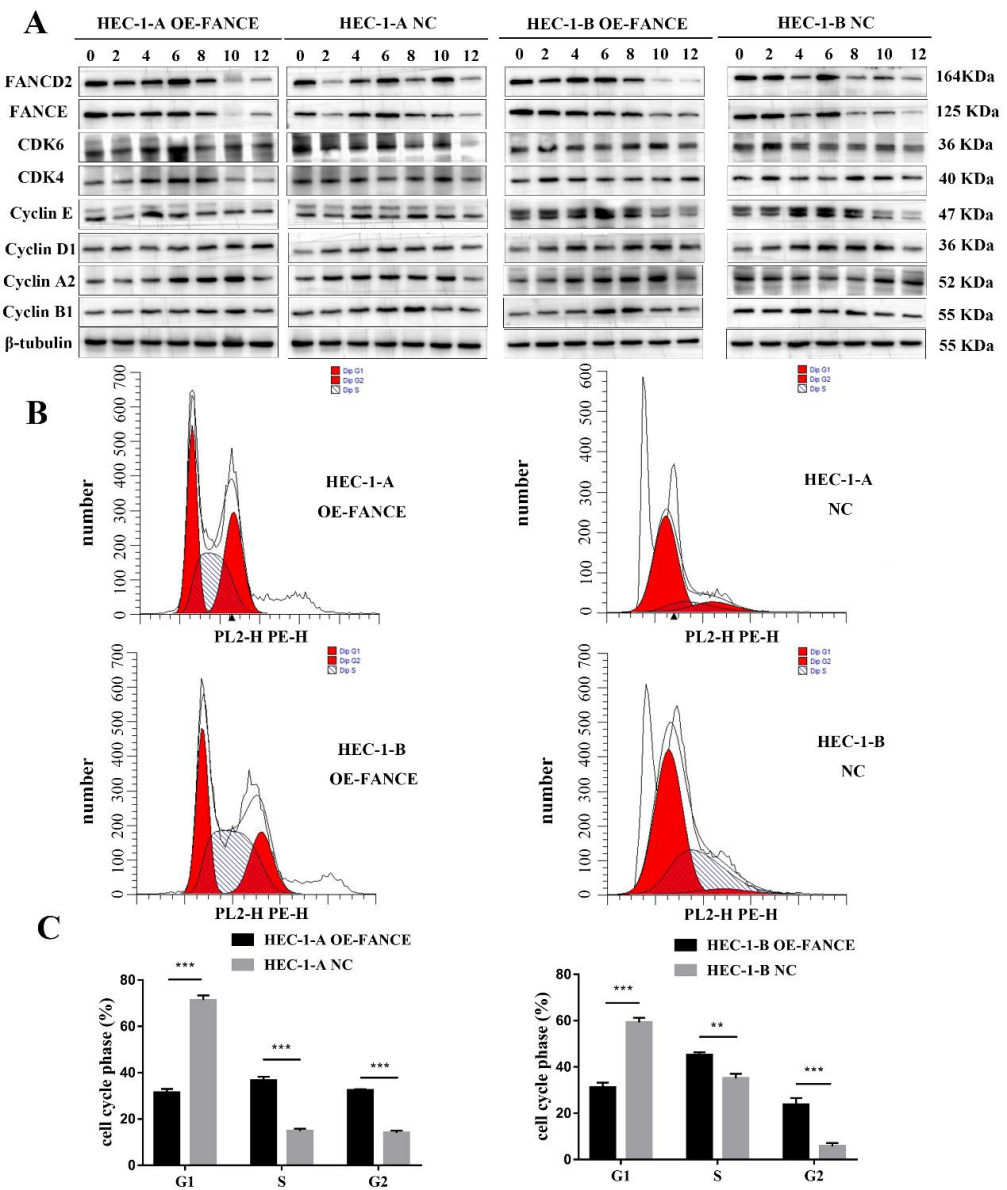
** FANCE suppressed cell cycle progression in EC cells** (A) The protein lysates of HEC-1-A OE-FANCE, HEC-1-B OE-FANCE, and corresponding NC cells were collected at the indicated time points after double-thymidine block and CDK4, CDK6, cyclinA2, cyclin B1, cyclin D1 and cyclin E expression were detected by WB. β-tubulin was used as the housekeeping protein. FANCE induced G2/M arrest and delayed entry to mitosis. (B) Analysis of EC cell cycle distribution with flow cytometry. (C) The proportion of cells in G1 phase was decreased and S and G2 phase was increased in HEC-1-A OE-FANCE and HEC-1-B OE-FANCE cells. ** p < 0.01, *** p < 0.001.

To determine the molecular regulation of FANCE in EC cells, cells were synchronized in early S phase and then released, and then cell cycle progression was examined by WB. The delayed upregulation of cyclin A2 in HEC-1-A OE-FANCE and HEC-1-B OE-FANCE cells indicated the prolongation of S phase. Compared to HEC-1-A NC and HEC-1-B NC cells, HEC-1-A OE-FANCE and HEC-1-B OE-FANCE cells exhibited delayed upregulation of Cyclin B1, indicated G2/M arrest and delayed entry to mitosis. As cells further progressed through the cell cycle, delayed increased Cyclin D1-CDK4/6 indicated delayed initiation of a new cell cycle round (Fig. 6A). Furthermore, flow cytometry analysis suggested significantly decreased cells in the G1 phase and increased cells in S and G2 phase (Fig. 6B, C). The results indicated G2/M arrest and mitosis delay in HEC-1-A OE-FANCE and HEC-1-B OE-FANCE cells.

### FANCE was involved in the regulation of multiple pathways in EC cells

**Figure 7:**
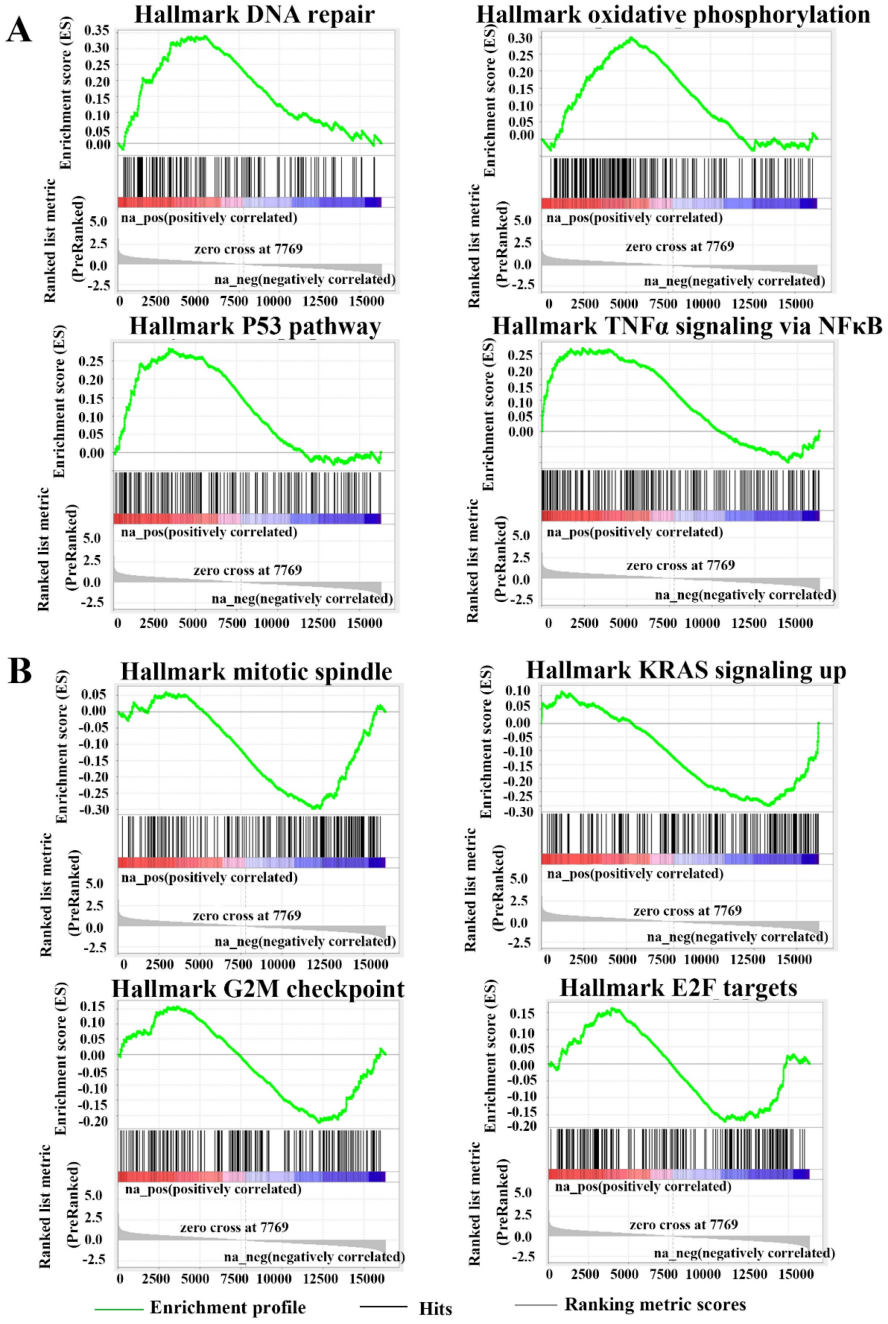
** FANCE was involved in the regulation of multiple pathways in EC cells** (A) Enrichment gene sets in HEC-1-A OE-FANCE cell as compared with HEC-1-A NC cell via Gene Set Enrichment Analysis. Up-regulated pathways: DNA repair pathway (FDR=0.002, p=0.000), oxidative phosphorylation (FDR=0.007, p=0.000), p53 pathway (FDR=0.015, p=0.000) and THFα signaling via NFκB pathway (FDR=0.026, p=0.003). (B) Down-regulated pathways: KRAS signaling up pathway (FDR=0.042, p=0.001), mitotic spindle pathway (FDR=0.051, p=0.000), G2/M checkpoint (FDR=0.427, p=0.159) and E2F targets (FDR=0.830, p=0.726).

RNA sequencing and gene set enrichment analysis revealed FANCE was involved in the regulation of multiple pathways in EC cells. GSEA enrichment analysis showed that DNA repair pathway (FDR=0.002, p=0.000), oxidative phosphorylation (FDR=0.007, p=0.000), p53 pathway (FDR=0.015, p=0.000) and THFα signaling via NFκB pathway (FDR=0.026, p=0.003) were significantly up-regulated in HEC-1-A OE-FANCE cells (Fig. 7A). KRAS signaling up pathway (FDR=0.042, p=0.001) and mitotic spindle pathway (FDR=0.051, p=0.000) were significantly down-regulated in HEC-1-A OE-FANCE cells, the latter was consistent with WB and flow cytometry results. Cell cycle-related G2/M checkpoint (FDR=0.427, p=0.159) and E2F targets (FDR=0.830, p=0.726) were downregulated in HEC-1-A OE-FANCE cells, while they were not significant (Fig. 7B).

### FANCE overexpression inhibited tumor growth *in vivo*

**Figure 8:**
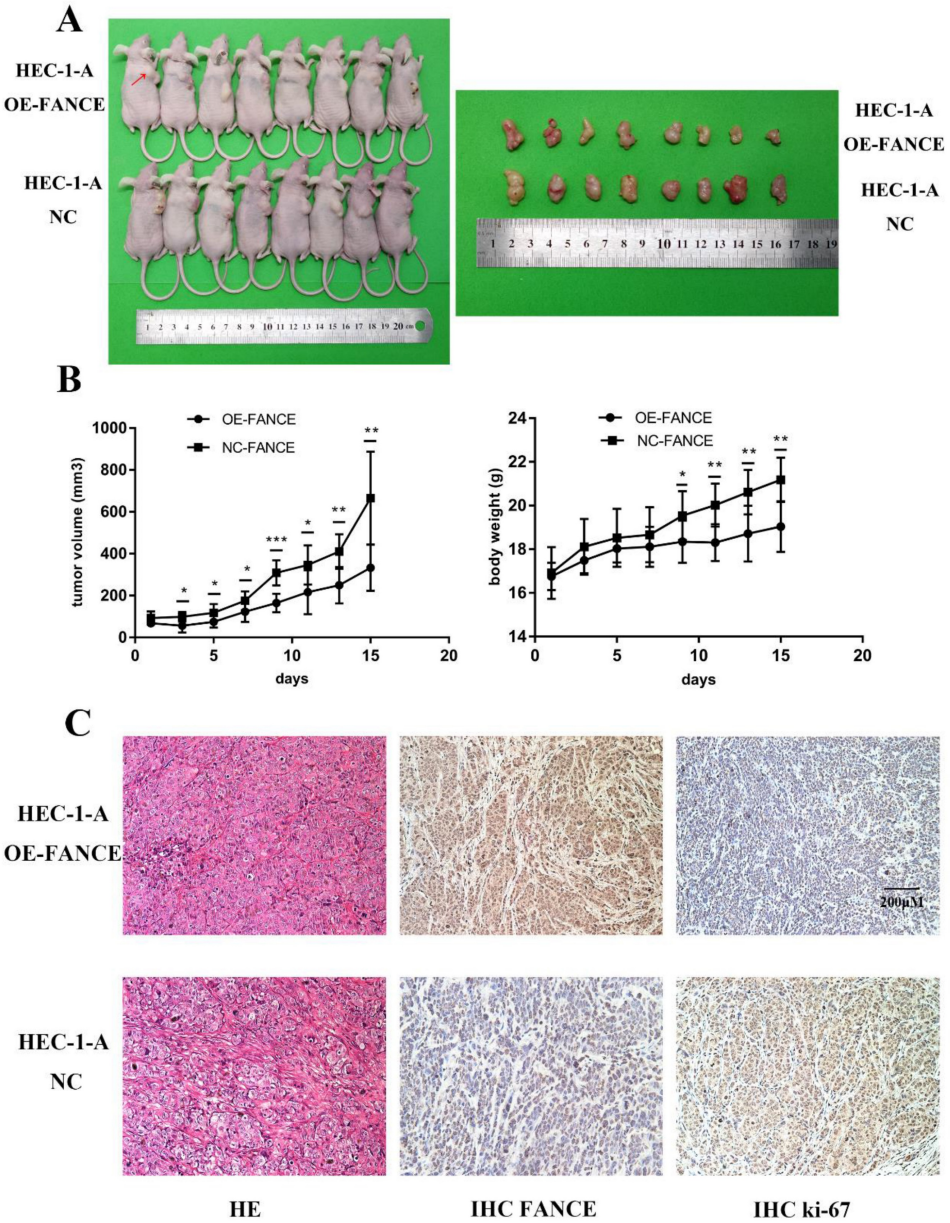
** Overexpression of FANCE inhibited mouse tumor growth *in vivo*** (A) Tumor formation with subcutaneous injection of HEC-1-A OE-FANCE or HEC-1-A NC cells. (B) The tumor volume and mice body weight were significantly reduced in HEC-1-A OE-FANCE group. (C) HE staining, IHC FANCE and ki-67 expression in isolated tumor tissue. Scale bar, 200μM. * p < 0.05, ** p < 0.01 and *** p < 0.001.

Furthermore, to verify whether our in-vitro results are valid *in vivo*, we constructed a nude mouse tumorigenesis model using HEC-1-A cells. As shown in Fig. 8, a significant reduction of tumor volume and tumor growth was observed from the third day in HEC-1-A OE-FANCE group. Likewise, the body weight of the mice began to drop from the ninth day. Finally, the tumor weight was significantly reduced in HEC-1-A OE-FANCE group compared to HEC-1-A NC group. H&E staining results showed EC-like tumor formation. Decreased Ki-67 expression was detected in HEC-1-A OE-FANCE compared to HEC-1-A NC group. Overall, FANCE overexpression inhibited EC tumor growth *in vivo*.

## Discussion

FANCE is a pivotal subunit of the FA core complex, promoting activation of the FA pathway and maintaining genome stability in response to multiple genotoxic stresses. Monoallelic germline mutations in some FA genes significantly increase susceptibility to adult-onset solid cancers but do not cause FA^20^. The Cancer Genome Atlas (TCGA) computational analysis demonstrated that somatic genetic alteration of FA genes is widely detected in multiple cancer types. FA genes were altered in 40% of nine common solid cancer types involved ovarian cancer and endometrial cancer. Among them, FA core complex gene alteration accounted for 9.9%^21^. Generally, deletions and loss-of-function mutations of FA genes induce genomic instability responsible for malignant transformation and cancer progression, but simultaneously they confer sensitivity to DNA damage agents. FANCE alterations have been previously reported to play a role in ESCC and HCC^13^, ^15^. However, less is known about FANCE in the occurrence and progression of EC. Our analysis showed that FANCE was significantly downregulated in EC and mediates cellular genomic instability, increasing sensitivity to cisplatin and MMC. We further used *in vitro* and *in vivo* functional assays to confirm that low-FANCE expression was associated with increased malignant proliferation and metastasis. Our study is the first time to demonstrate the role of FANCE in the initiation and progression of EC.

In our study, the RNA and protein expression of FANCE in fresh frozen specimens and paraffin sections of EAC and noncancerous tissues were comprehensively detected by RT-PCR, WB and IHC assays. FANCE was significantly downregulated in EC but did not appear to be associated with progression of disease transformation, as normal endometrium and AEH were not significantly different. The results suggest that low expression of FANCE is tumor heterogeneity. Correlation analysis of clinical parameters showed that decreased FANCE expression was associated with older age (p=0.021) and higher tumor grade (p=0.035). Age at diagnosis and histological grade were predictive factors for survival 22. The low expression of FANCE indirectly reflects the malignant behavior of EC. Since the patients are all new cases from 2020 to 2021, the relationship between FANCE and clinical outcomes needs to be further supplemented.

We used alkaline comet assay and fluorescence staining of γH2AX to show genetic stability effect of OE-FANCE in EC cells. We examined BLEO-induced DNA single- and double-strand breaks by measurable comet tail moment^23^, and found cisplatin-induced ICL formation inhibited this process^24^. Our results indicates that FANCE is involved in the repair of ICLs damage, and EC OE-FANCE cells form fewer DNA ICLs. Cellular DSB and ICL damage can induce γH2AX foci formation^25^, EC OE-FANCE cells showed decreased γH2AX expression. We demonstrated through direct and indirect methods that FANCE promotes cellular DSB repair and ICL repair. The chemosensitivity of EC OE-FANCE cells to cisplatin and MMC treatment was reduced, consistent with the mechanism of action of the FA pathway. Synthetic lethality could theoretically be achieved by exploiting the high reliance on compensatory DNA repair caused by defects in the FA pathway^26^, which may aid therapeutic exploration in EC. The efficacy of poly-ADP ribose polymerase (PARP) inhibitors in EC patients with reduced or absent FANCE expression is worthy of further investigation.

In addition, FA proteins are involved in cell cycle regulation^27^. Deletion of FANCC in liver cancer cell lines resulted in cells exhibiting sensitivity to ICL agents and G2 cell cycle arrest^28^. Depletion of FANCD2 protein expression significantly inhibited ESCC cell proliferation and metastatic potential, as well as cell cycle progression, by involving cyclin-CDK and ATR/ATM signaling^29^. Our data suggested that FANCE inhibits cell proliferation through G2/M cell cycle arrest. Moreover, RNA sequencing confirmed these results. The limitation is the role of FANCE in other signal transduction pathways requires further investigation.

In summary, our study demonstrates that FANCE expression is significantly downregulated in EC. The reduction of FANCE expression leads to genomic instability, thereby promoting the occurrence and development of EC. Overexpressing FANCE inhibits cell proliferation and -invasion by arresting cell cycle progression. These observations provide new evidence for the regulation of DNA damage repair by FA pathway in EC.

## Figures and Tables

**Table 1:** Comparison of FANCE and clinical parameters of EAC, AEH and normal endometrium

Clinical parameters	EAC (n=83)	AEH (n=30)	Normal endometrium (n=83)	P value
Age <55 ≥55	36 (43.4%) 47 (56.6%)	11 (36.7%) 19 (63.3%)	40 (48.2%) 43 (51.8%)	0.536
Hypertension Yes No	14 (16.9%) 69 (83.1%)	5 (16.7%) 25 (83.3%)	9 (10.8%) 74 (89.2%)	0.532
Diabetes Yes No	10 (12%) 73 (88%)	3 (10%) 27 (90%)	8 (9.6%) 75 (90.4%)	0.95
Menopause Yes No	55 (66.3%) 28 (33.7%)	16 (53.3%) 14 (46.7%)	40 (48.2%) 43 (51.8%)	0.063
Family history of malignancy Yes No	10 (12%) 73 (88%)	3 (10%) 27 (90%)	4 (4.8%) 79 (95.2%)	0.271
FANCE level FANCE-high FANCE-low	41 (49.4%) 42 (50.6%)	24 (80%) 6 (20%)	72 (86.7%) 11 (13.3%)	#0.000

EAC, endometrioid adenocarcinoma. AEH, endometrial hyperplasia. FANCE, fanconi anemia complementation group E. p=#0.000 represents the statistical difference between EAC and AEH, EAC and normal endometrium.

**Table 2:** Correlation between FANCE expression and EAC clinicopathological features

Clinicopathological features	FANCE-high expression (n=41)	FANCE-low expression (n=42)	P value
Age <55 ≥55 FIGO grade	22 19	14 28	0.021
1 2	28 11	20 12	0.035
3	2	10	
FIGO stage			
I	32	29	
II	5	5	0.474
III	4	8	
Depth of MI			
≤1/2	30	29	0.679
>1/2	11	13	
Lymph node positive			
No	37	39	0.713
Yes	4	3	
LVI			
No	37	36	0.767
Yes	4	6	
MMR			
pMMR	32	27	0.167
dMMR	9	15	
Menopause			
yes no	23 18	32 10	0.053
Ki-67 positive negative P53 positive negative	17 24 25 16	14 28 25 17	0.444 0.893

FANCE, fanconi anemia complementation group E. FIGO, Federation of Gynecology and Obstetrics. MI, myometrial invasion. LVI, lympho-vascular involvement. MMR, mismatch repair. P53, protein 53.

## Abbreviations

EC: endometrial cancer
EAC: endometrioid adenocarcinoma
HR: homologous recombination
DDR: DNA damage repair
FA: fanconi anemia
ICL: interstrand cross-link
FANCE: fanconi anemia complementation group E
FANCD2: fanconi anemia complementation group D2
ESCC: esophageal squamous cell carcinoma
HCC: hepatocellular carcinoma
AEH: atypical endometrial hyperplasia
MI: depth of myometrial invasion
LVI: lympho-vascular involvement
MMR: mismatch repair
FIGO: International Federation of Gynecology and Obstetrics
IHC: immunohistochemistry
H&E: hematoxylin and eosin
DAB: 3,3-Diaminobenzidine
overexpressing FANCE: OE-FANCE
NC: negative control
siRNA: small interfering RNA
qRT-PCR: quantitative real-time polymerase chain reaction
GAPDH: gene glyceraldehyde 3-phosphate dehydrogenase
WB: western blotting
RIPA: radio immunoprecipitation assay
PVDF: polyvinylidene fluoride
BSA: bovine serum albumin
RAD51: RAD51 homolog 1
γH2AX: gamma H2A histone family member X
CDK4: cyclin dependent kinase 4
CDK6: cyclin dependent kinase 6
ECL: Enhanced Chemiluminescent
CCK8: Cell-Counting-Kit-8
OD: optical density
IC50: inhibitory concentrations 50%
MMC: mitomycin C
EDU: 5-ethynyl-2'deoxyuridine
PBS: phosphate buffer saline
PI: propidium iodide
DAPI: 4',6-diamidino-2-phenylindole
BLEO: bleomycin sulfate
SD: standard deviation
DSB: double-strand break
FANCE-KD: FANCE-knockdown
GSEA: gene set enrichment analysis
FDR: false discovery rate
TCGA: The Cancer Genome Atlas
PARP: poly-ADP ribose polymerase
